# Supplementary material for: Are carbon emissions trading and green financial instruments synergistic? -Comprehensive quantitative research based on content analysis
Source: PLoS One. 2024 Mar 7;19(3):e0298601. doi: 10.1371/journal.pone.0298601 (PMC10919601; doi:10.1371/journal.pone.0298601)
Supplement: S1 Data — (ZIP) [file pone.0298601.s001.zip › Data/地级市Z_F工作报告.zip!/║■─╧/│╗╓▌╩╨/└┤╫╘╣1⁄2╓┌║┼í╛┬φ┐╦╩2╛▌═°í┐_2017.docx]

各位代表：

现在，我代表市人民政府向大会报告过去五年的工作，对今后五年及2017年的工作提出建议，请予审议，并请市政协委员及其他列席人员提出意见。

一、过去五年的工作回顾

刚刚过去的2016年，是“十三五”的开局之年。一年来，在省委、省政府和市委的坚强领导下，在市人大的法律监督、工作监督和市政协的民主监督及大力支持下，市人民政府紧密团结和依靠全市人民，认真贯彻落实党的十八大及十八届三中、四中、五中、六中全会精神，坚持稳中求进工作总基调，坚定发展信心，克服各种困难，大力实施“产业主导、全面发展”战略，扎实推进以落实四个“四年行动计划”为重点的各项工作，全力以赴兴产业、强实体、提品质、增实效，实现了“十三五”良好开局。预计（下同），全市地区生产总值增长8.3%；一般公共预算收入增长6.9%，地方财政收入增长7.7%；固定资产投资增长14.5%；社会消费品零售总额增长11.7%；实际利用外资增长10%；城镇、农村居民人均可支配收入分别增长8.7%、9%；城镇登记失业率控制在3.54%以内；社会事业全面发展，各项工作取得新的进步。在建设“五个郴州”、打造湖南“新增长极”、率先全面建成小康社会的征程中，我们迈出了坚实的一大步。

各位代表！过去五年，虽然宏观经济形势复杂多变、经济下行压力持续增大，但在市委的正确领导下，全市上下始终紧紧抓住发展第一要务不动摇，主动适应引领经济发展新常态，坚持不懈打基础、抓关键、补短板，扎实抓好“一化两城三创四大”“产业主导、全面发展”战略和“五个郴州”建设，全力推进产业转型、动能转换、作风转变，一批标志性、引领性的重大工程项目相继实施或竣工，一批国家或省级层面的重大规划、试点试验稳步推进，一批新的经济增长点正在加快形成，是郴州综合实力提升最快、城乡面貌变化最大、创新创业活力最强、群众得到实惠最多的时期之一。全市地区生产总值、规模工业增加值、一般公共预算收入总量分别迈上2000亿元、1000亿元、200亿元台阶，年均增长10.3%、10.8%、9.3%。北湖、桂阳、资兴、永兴等4个县市区生产总值突破300亿元。全市全面小康实现程度由75.8%提高到93%，资兴、永兴、桂阳、苏仙进入全省首批20个全面小康达标县市区之列。

五年来，我们突出质量效益兴产业、强实体，郴州综合实力显著增强。传统产业转型升级。有色金属“五个一”战略体系基本建成，21家有色金属企业进入全省行业50强，有色金属产业成为全市首个千亿产业。机械制造等传统工业通过技术创新、产品换代焕发新的生机。嘉禾被授予“中国锻造之乡·绿色锻造发展基地”。煤矿、非煤矿山分别减少104个、129个。观光旅游加速向休闲旅游转变，景点旅游加速向全域旅游转型，成功创建国家级休闲城市，新增国家5A级景区1个、4A级景区5个；桂东、苏仙、资兴、汝城、宜章等5个县市区列入国家全域旅游示范区创建单位；接待游客人数、旅游综合收入年均分别增长16.4%、19.4%。金融业蓬勃发展，金融机构存贷款余额分别增加1182亿元、600亿元。金贵银业成功上市，高斯贝尔首发申请顺利通过证监会发审委审核，金晋牧业、格兰博科技、金煌物流在“新三板”挂牌。房地产业持续健康发展，完成去库存310万平方米，旅游地产、养老地产等新业态逐步兴起。成功获批国家“十三五”服务业综合改革试点。粮食产量保持稳定。农村一、二、三产业加快融合，休闲农业、庄园经济方兴未艾。国家现代农业示范区和国家农业科技园区成功创建。汝城、桂东跻身全国重点产茶百强县。新兴产业迅速崛起。新能源产业迅猛发展，风力发电装机占全省总量的近一半。电子信息产业加速壮大，LED产业列入省级创新型产业集群。先进装备制造、新材料、生物制药等产业势头看好。精品会展和宝玉石产业迅速发展，通天玉湖南省地方标准实施，连续举办2届矿博会、4届湘南投洽会、2届湖南特色农博会，郴州被确定为中国（湖南）国际矿物宝石博览会固定举办地，并成为全省会展次中心。成功承办一系列大型国际体育赛事，被列为国家体育产业首批试点城市。阿里巴巴等知名电商进驻郴州，成功创建国家电子商务示范市。现代物流业加快发展，新增2家5A级现代物流企业。战略性新兴产业增加值占GDP的比重提高到19%。园区经济加快壮大。创新创业园区“1555”工作计划全面完成，园区规模工业增加值增长2.6倍，占全市规模工业增加值的76.9%。郴州高新区升格为国家级高新区并跻身“千亿园区”行列，郴州出口加工区升级为综合保税区。

五年来，我们坚持项目带动抓投资、促消费，郴州发展基础不断夯实。累计完成固定资产投资9039亿元。衡茶吉铁路、厦蓉等3条高速公路、郴永大道、郴州国际会展中心、江源水库、沤菜水库、华瀚科技、中化弘源化工、国际矿产资源交易中心、南方矿业技改、金贵白银城等一批重大项目竣工；东江引水工程、莽山水库、桃源水库、高铁西站扩容提质、郴州卷烟厂易地技改、东江湖大数据产业园、飞天山文化旅游产业园、长鹿国际旅游休闲度假区等一批重大项目进展顺利；桂新高速公路等重大项目前期工作取得突破性进展，郴州北湖机场已经国务院、中央军委批复同意并启动配套项目建设，赣郴永兴铁路纳入国家促进中部崛起“十三五”规划。市场建设成效显著，一批大型专业市场和城市综合体建成营业。住房消费保持稳定增长。累计完成棚户区改造6.88万套、农村危房改造10.42万户。社会消费品零售总额年均增长13.4%。消费对经济增长的贡献率年均超过48%。

五年来，我们深化改革创新破瓶颈、除障碍，郴州开放活力、创新动力加快迸发。全面深化改革取得系列突破。按照“四个有利于”要求，着力攻克政府机构改革、行政审批提速、行业协会脱钩、公共资源交易平台整合等一批“硬骨头”，行政审批和商事登记制度改革、生态文明制度建设、投融资体制改革、国企改革、农村综合改革等走在全省前列，国有林场改革、乡镇区划调整、建制村合并全面完成，全省改革“先行区”的地位继续巩固。创新驱动发展战略成效明显。稀贵金属深加工、石墨高端产品研发、电子信息技术创新等高新技术取得突破，专利授权量年均增长15.1%，获国家科技奖励3项，被列为国家知识产权示范培育城市。新增3家国家创新平台、5家院士工作站、1家国家级质检中心，培育中国驰名商标和国家地理标志保护产品17件。高新技术产业增加值增长近2倍。对外开放水平持续提升。海关、口岸、检验检疫等开放平台进一步完善，纳入广东往来港澳陆路跨境快速通关改革试点市，跻身“无水港”城市行列。累计新建标准厂房1541万平方米，引进转移型企业1000余家，实际利用外资58.2亿美元，外贸进出口总额165.9亿美元。深圳正威、北京顺峰、广东长鹿等一批战略投资者落户郴州。开放包容的郴州，发展要素集聚能力明显增强。

五年来，我们围绕协调发展抓统筹、补短板，郴州生态品牌全面创响。统筹城乡全面发展，推进生态文明建设，在厚植优势、补齐短板中不断提升了发展的平衡性、包容性、可持续性。城乡格局发生巨变。市城区在全市城镇体系中的龙头地位更加突出，郴资桂-郴永宜“大十字”城镇群加快融合，县城和示范镇、特色镇建设加快推进，全市城镇化率由43.3%提高到51.3%。郴州市、资兴市被列为全国新型城镇化综合试点，永兴、嘉禾、宜章、安仁列入国家智慧城市试点县。新建高速公路404公里，国省干线公路881公里，农村公路2779公里，100%的行政村通水泥路。城乡客运一体化进程加快。公路“治超”持续巩固深化。城乡品质显著提升。市城区累计投入基础设施项目建设资金700亿元，南岭大道、塘尾大道、卜里坪路、温泉路等建成通车，建成人行天桥12座、地下通道15处，背街小巷、农贸市场改造基本完成。郴江河、燕泉河综合治理全面竣工。“公交都市”加快创建。全市新投放新能源公交车1300余台。新增管道燃气用户12.3万户。城市精细化管理水平明显提升，成功创建国家园林城市、国家卫生城市、全国交通管理模范城市，获得全国文明城市提名资格。汝城成功创建国家园林县城。水利建设投资200亿元，治理病险水库780座。切实加强全市饮用水安全保障工作，解决了150.5万农村人口的饮水安全问题。“点亮郴州”实现全覆盖，规范农村村民建房、农村环境整治取得显著成效。总长140公里的西河生态休闲风光带全面建成。郴州获评“中国美丽乡村建设示范地区”。生态质量明显改善。切实加强东江苏仙段（翠江）流域环境污染综合治理，并取得阶段性成果，流域内水环境质量明显好转。湘江保护和治理纵深推进，“一湖两河三江”生态综合治理启动实施。6个县市列为国家主体生态功能区国家级示范试点。全国水生态文明城市建设试点任务全面完成。东江湖入选国家湖泊生态环境保护重点支持湖泊。全市森林覆盖率67.71%。新增安仁熊峰山、嘉禾南岭等6个国家森林公园和郴州西河等3个国家湿地公园。成功创建国家森林城市、全国绿化模范城市，列入全国生态文明示范工程试点市，入选中国50大“氧吧”城市。一张张“国字号”生态名片，极大地提升了郴州的知名度、美誉度。

五年来，我们聚焦群众关切办实事、惠民生，人本郴州发展理念深入人心。始终坚持以人民为中心的发展思想，持续实施“民生100工程”和重点民生实事项目，民生领域支出累计1286亿元，每年财政民生支出占一般公共预算支出比重保持在75%以上；累计新增城镇就业34.2万人、农村劳动力转移就业23.9万人，荣获“全国创业先进城市”称号；城镇、农村居民人均可支配收入分别达27576元、12838元，年均增长10%、12.2%；按照“七个精准”“五个一批”要求，扎实推进精准扶贫、精准脱贫，累计脱贫36.73万人。社会保障水平持续提升，实现城乡居民医疗保险一体化管理，建立了统一的城乡居民基本养老保险制度和大病保险制度。各类教育协调发展，创建村小合格学校974所、县城以上城区新增义务教育学位15.14万个，化解大班额取得显著成效。开展“教育突出贡献奖”评选表彰，全市教育教学质量稳步提升。郴州师范升格为湘南幼儿师范高等专科学校，郴州职业技术学院建成省示范性（骨干）高职院。公共文化服务体系进一步完善，农家书屋、农村广播“村村响”实现行政村全覆盖。历史文化名城保护与发展切实加强，成功申报国家级历史文化名村1个、中国传统古村落35个、省级历史文化名村13个，一大批重要文物和非物质文化遗产得到进一步保护。“体育之城”创建取得阶段性成果，郴籍运动员易思玲、史逸婷分别在伦敦奥运会和里约残奥会获得1枚金牌。医药卫生体制改革稳步推进，公共卫生、计生服务均等化水平明显提升。实施全面两孩政策，人口自然增长率7.31‰，出生人口素质有效提高、人口结构不断优化。民族宗教、外事侨务、对台事务、统计、物价、人防、档案、气象、地震、水文、科普、地方志和妇女儿童、老年人、残疾人、慈善等事业取得新进步。驻郴解放军、武警、消防部队和民兵预备役为地方发展作出新贡献，军民融合发展深入推进。安全生产形势持续稳定向好。信访维稳、食品药品安全、防汛抗灾、森林防火等工作成效显著。平安创建扎实开展，普法依法治理工作稳居全省前列，连年获评“全省社会管理综合治理先进市”。人民群众的安全感、获得感、幸福感与日俱增，入选“中国最具幸福感的30座城市”。

五年来，我们认真依法履职转作风、优服务，政府执行能力明显提高。在市委的坚强领导下，认真执行市人大及其常委会的决议决定，自觉接受市人大的法律监督、工作监督和市政协的民主监督。坚持以提高质量和满意率为核心，扎实推进人大代表建议、政协提案办理工作，共办理市人大代表建议1238件、市政协提案1257件，办复率均为100%。建立重大行政决策合法性审查机制和法律顾问制度，开展规范性文件合法性审查525件，组织重大行政决策听证37场次、专家论证18场次，聘请政府法律顾问3批23人次，政府科学决策机制不断完善，行政权力运行进一步规范，法治政府建设稳步推进。坚持简政放权、放管结合、优化服务，行政处罚电子监察系统、投资项目在线审批监管平台全面建成，商事登记“五证合一、一照一码”全面实施，“三清单一目录”全面落实，非行政许可全面取消，市县乡三级政务服务体系基本建成，行政效能和服务水平明显提升。坚持人民至上，强化责任担当，着力解决了一批与群众利益直接相关的历史遗留问题。注重发挥各民主党派、工商联、无党派人士的参政议政作用，支持工会、共青团、妇联、计生协等群团组织充分发挥桥梁纽带作用。坚定不移推进全面从严治党，强化“四个意识”，落实“两个责任”，扎实开展党的群众路线教育实践活动、“三严三实”专题教育和“两学一做”学习教育，持续开展“作风大整顿大优化大提质”行动和“雁过拔毛”式腐败问题专项整治，群众反映强烈的突出问题得到及时有效解决。以“零容忍”态度惩治腐败，严查违法违纪案件，人民群众对政府的满意度不断提高。

各位代表！五年来，全市人民弘扬“开放包容、求实创新”的郴州精神，一起挥洒汗水，一路同甘共苦，应对了一次次挑战，办成了一件件实事，实现了一个个梦想。特别是在交通建设、城市提质、产业转型、城乡统筹、生态建设、社会治理、民生保障、简政放权、作风建设等很多方面，创造了“郴州速度”，创响了“郴州品牌”，产生了“郴州现象”。成绩的取得，是省委、省政府和市委正确领导、科学决策的结果，是市人大、市政协监督支持、积极参与的结果，是全市人民团结拼搏、务实进取的结果，更体现了强化“郴州担当”、凝聚“郴州力量”、凸显“郴州实效”的丰硕成果。在此，我谨代表市人民政府，向全市各族人民，向人大代表、政协委员，向各民主党派、工商联、无党派人士和各人民团体，向离退休老同志，向驻郴部队官兵和中省驻郴单位，向所有关心支持郴州改革发展的各级领导、各界朋友，表示衷心的感谢并致以崇高的敬意！

总结过去五年，我们深刻体会到：做好政府工作，必须坚持以党的领导为根本保证，牢固树立“四个意识”，坚决维护以习近平同志为核心的党中央权威，坚定正确的政治方向，坚守政治纪律和政治规矩，坚决贯彻党的路线方针政策，认真落实省委、省政府和市委决策部署，在强化执行、狠抓落实中攻坚克难；必须坚持以改革创新为根本动力，把创新作为引领发展的第一动力，勇于解放思想、先行先试，敢于探索实践、改革创新，用改革的办法破解难题，用创新的力量驱动发展，在改革创新中不断开拓前进；必须坚持以开放发展为根本途径，主动对接国家战略，以国际化现代化的视野，聚焦“珠三角”、放眼全世界，坚持对外开放与对内开放相结合、“引进来”与“走出去”相结合、引资与引智引技相结合，在开放中拓展发展空间、提升发展水平、增强发展后劲；必须坚持以绿色生态为根本要求，坚持“绿水青山就是金山银山”，树牢绿色发展理念，守住生态文明底线，坚定走生产发展、生活富裕、生态良好的发展道路，不断满足人民群众对清新空气、干净饮水、安全食品、优美环境的需求；必须坚持以服务人民为根本宗旨，始终把人民对美好生活的向往作为我们的奋斗目标，不忘初心、践行宗旨，全力办好民生实事，让人民群众日子过得更顺心、更舒心、更称心。

同时，我们也清醒看到工作中存在的困难和问题，主要是：对经济新常态的趋势性变化认识不够，在推动工作落实上办法还不够多、措施还不够有力，地区生产总值、一般公共预算收入等部分主要经济指标没有实现市四届人大一次会议确定的目标；支撑经济发展的大产业、大企业、大项目不多，自主创新能力不强，动能转换不快，转型发展仍然任重道远；财政收入质量还不高，财源建设亟待加强；居民持续增收难度加大，改善民生和脱贫攻坚任务艰巨；自然生态系统和环境基础脆弱，环境保护和安全生产形势仍然严峻；腐败以及少数干部进取精神不强、“懒散庸”现象依然存在，创新政府公共服务和社会治理刻不容缓等。我们将采取有效措施，切实解决这些问题。

二、今后五年的总体思路和工作目标

今后五年，是郴州深化创新引领、加快开放崛起的关键时期，是在全省率先完成贫困片区脱贫摘帽、在湘南率先全面建成小康社会的关键时期。随着国家“一带一路”“长江经济带”“一片一区”等战略，全省“一带一部”“一核三极四带多点”区域发展战略，以及市委“产业主导、全面发展”战略的深入实施，多重利好同步释放。特别是，通过大力推进创新开放发展，新的经济增长点正在加快形成，郴州发展的韧性和后劲极大增强，后发优势将更加明显。我们有条件、有基础，在抢抓战略机遇中发展得更好。

根据省第十一次党代会和市第五次党代会精神，今后五年政府工作的总体思路是：高举中国特色社会主义伟大旗帜，以马克思列宁主义、毛泽东思想、邓小平理论、“三个代表”重要思想、科学发展观为指导，认真贯彻落实党的十八大及十八届三中、四中、五中、六中全会精神和习近平总书记系列重要讲话精神，按照“五位一体”总体布局、“四个全面”战略布局和“五大发展理念”总要求，大力实施“创新引领、开放崛起”和“产业主导、全面发展”战略，统筹推进经济建设、政治建设、文化建设、社会建设和生态文明建设，加快建设实力郴州、创新郴州、开放郴州、生态郴州、人本郴州，致力打造湖南“新增长极”，为率先全面建成小康社会而努力奋斗。

加快建设“五个郴州”、打造湖南“新增长极”、率先实现全面小康，是市第五次党代会确定的未来五年全市发展的总任务。抓好这一总任务的落实，是未来五年政府工作的核心。具体来说，要通过狠抓落实，努力实现以下六个方面的目标。

——健全现代产业体系，在建设实力郴州上实现重大突破。坚持产业主导、全面发展，着力培育和引进一批核心竞争力强、主业优势明显、关联带动突出的大企业；强化产业公共服务体系建设，推动产业结构迈向中高端，加快构建具有较强竞争力的现代产业体系，着力培育一批千亿产业和园区。全力打造“林中之城、休闲之都”“碧水丹霞、福地郴州”“矿物宝玉、精品博览”三大品牌和全国重要的新型有色金属产业基地、全域旅游基地、石墨及新材料产业基地、绿色生态产业基地、粤港澳优质农产品供应基地等五大基地。力争地区生产总值突破3000亿元，一般公共预算收入突破300亿元。

——健全要素供给体系，在建设创新郴州上实现重大突破。落实“四个有利于”要求，更加精准、务实、有效地全面深化改革，确保重点领域和关键环节改革继续走在全省前列。坚持创新引领，整合优质创新资源向重点领域、重点产业、关键环节倾斜，着力打造全要素、开放式的创新创业生态系统。积极争取以“3+1”模式纳入“长株潭”国家自主创新示范区建设范围，成为全省重要的创新型城市。扎实推进全国新型城镇化综合试点工作，积极探索加快新型城镇化进程的体制机制，增强“大十字”城镇群辐射带动能力，形成梯次错位发展、多点多极支撑的新型城镇体系，加快建设一批产镇融合、产城一体的国家级、省级特色小镇，力争城镇化率达到全国平均水平。始终重视“三农”工作，创新“三农”工作理念、思路和举措，促进城乡一体化发展。

——健全对外开放体系，在建设开放郴州上实现重大突破。进一步解放思想，扩大对内对外开放，抓实平台建设、产业招商和区域合作，走出一条更高层次的开放发展之路，努力打造湖南开放崛起的“桥头堡”。全力推进综合立体交通建设，力争赣郴永兴铁路和郴资永轨道交通开工建设，确保郴州北湖机场2019年建成通航，力争打造通用航空示范市，基本建成区域性立体化综合交通枢纽城市，致力建设区域特色会展中心、全国有色金属交易中心、省际区域医疗卫生中心、省际边界商贸物流中心。

——健全生态承载体系，在建设生态郴州上实现重大突破。坚持生态优先、绿色惠民、低碳发展，实行最严格的生态保护制度。大力开展大气、水、土壤污染防治行动，全面实施山水林田湖生态保护和修复工程，筑牢生态安全屏障，构建全域覆盖的生态环保体系，推动形成绿色发展方式和生活方式，全面完成节能减排任务，为子孙后代留下天蓝、地绿、水碧、土净的生产生活环境。

——健全民生保障体系，在建设人本郴州上实现重大突破。城镇、农村居民人均可支配收入分别突破4万元、2万元，确保2019年提前一年在扶贫片区率先脱贫摘帽、在湘南地区率先全面建成小康社会。城镇登记失业率控制在4.5%以内，社会保障体系更加健全。加快推进教育强市、文化强市和健康城市建设，促进教育、医疗卫生、人口计生、公共文化和体育等社会事业全面发展，补齐农村特别是贫困地区基础设施和公共服务短板。深化“平安郴州”建设，加强和创新社会治理，推进社会治理精细化、信息化、法治化，使人民群众的安全感、获得感、幸福感持续增强。

——健全行政权力运行和监督体系，在建设法治政府、强化决策执行上实现重大突破。贯彻落实中共中央、国务院《法治政府建设实施纲要（2015-2020年）》，深入推进简政放权、放管结合、优化服务，健全行政权力运行和监督体系，进一步强化行政效能建设，促进政风行风转变，基本建成职能科学、权责法定、执法严明、公开公正、廉洁高效、守法诚信的法治政府。健全完善考核激励机制，强化责任担当，狠抓工作落实，不断提高政府执行力和公信力。

各位代表！发展的蓝图已经描绘，肩负的使命非常神圣。只要我们切实增强机遇意识、责任意识、进取意识，立足“桥头堡”优势，担当“增长极”使命，奋发有为，扎实工作，就一定能够开创郴州更加辉煌的明天！

三、2017年主要工作任务

2017年，是全面贯彻落实省第十一次党代会和市第五次党代会精神的第一年，是供给侧结构性改革的深化之年，也是新一届市政府履职的第一年。根据中央、省委和市委经济工作会议精神，做好今年的政府工作，要坚持稳中求进工作总基调，坚持以提高质量和效益为中心，坚持以推进供给侧结构性改革为主线，坚持全面实施四个“四年行动计划”，坚持全面提升“六大产业”整体素质，扎实做好稳增长、促改革、调结构、惠民生、防风险各项工作，促进经济平稳健康发展和社会和谐稳定，以优异成绩迎接党的十九大召开。主要预期目标是：地区生产总值增长8.5~9%；规模工业增加值增长8%；一般公共预算收入和地方财政收入均增长7%；固定资产投资增长16%；社会消费品零售总额增长12%；外贸进出口总额增长10%；实际利用外资增长10%；城乡居民人均可支配收入增长8%；城镇登记失业率控制在4.5%以内。具体来说，要围绕实现上述目标，扎实抓好八个方面的工作：

（一）坚持转型升级，优化产业结构。结合推进工业转型升级、服务业发展两个“四年行动计划”，扎实开展“互联网+”行动，促进工业化与信息化、互联网与实体经济深度融合，加快供需结构优化升级。着力推进“三去一降一补”任务落实。加大煤矿关闭退出和整合力度，积极化解过剩产能。整顿关闭和淘汰不安全的落后企业，严控烟花爆竹企业数量。加快住房保障改革，全面推行棚改货币化安置，引导农民工市民化，发展房屋租赁市场，推进房地产去库存。加快政府融资平台市场化转型和存量债务置换，完善全口径政府债务管理，有效防范化解政府债务风险。全面深化政银企合作，强化金融对实体经济的支持，积极防范化解金融风险。全面落实各项减负政策，降低企业运行成本。着力推进传统产业转型升级。以发展精深加工为重点，深化有色金属“五个一”战略体系建设，支持永兴做大做强“中国银都”品牌，加快郴州稀贵金属供应链体系、国际矿产资源交易中心建设，推动全产业链发展。大力提升机械制造、食品等传统工业。支持嘉禾做大做强“江南铸都”品牌，推动传统铸锻造向中高端装备制造转型；支持临武城东、资兴罗围、北湖石盖塘、桂阳芙蓉等农产品工业园建设，扶持农业龙头企业发展壮大。围绕发展全域旅游，加强城市形象宣传，充分挖掘旅游投资和旅游消费潜力，大力实施“旅游文化千亿产业四年行动计划”，推动文化产业与旅游产业融合发展，积极创建“全国自驾游示范城市”，承办好2017中国（湖南）红色旅游文化节，以县市区为主体每季度举办一次乡村旅游文化节，支持宜章创建莽山国家5A级景区、汝城创建热水温泉国家级旅游度假区，加快建设旅游强市。提升发展金融服务业，引进更多金融机构入驻，创新金融产品和服务模式，完善农村金融体系和中小企业融资担保体系。大力推进农业现代化，加快三个“十百千”工程、精品农业庄园、现代农业示范区等农业项目建设，突出抓好湘南柑桔产业带、罗霄山脉茶叶产业带和粤港澳优质农产品供应基地建设，增加优质农产品供给。稳定发展烟草产业。继续开展“培育新型农业经营主体三年行动”，构建现代新型农业经营体系。加强农业服务体系建设和农作物病虫害监测防控、重大动物疫病防治。落实最严格的耕地保护制度，坚守耕地红线不动摇，实施耕地质量提升工程。稳定粮食总产190万吨左右。着力推进新兴产业发展壮大。立足资源优势和产业基础，重点发展稀有金属材料、新型复合材料、新能源材料、高性能合金材料、医药化工催化剂、智能金属产品等。深化与中国建材的战略合作，高标准规划建设石墨产业园，大力发展石墨提纯和精深加工。加快安仁精细化工园、中化蓝天郴州基地和氟化工产业园建设。抓好通天玉等宝玉石矿资源保护和开发，加快建设郴州矿物宝石产业园，培育引进一批宝玉石加工企业，筹建省级宝玉石检验检测中心，提升“金玉郴州”品牌效应。支持电子信息骨干企业发展，加快形成百亿产业集群。加快东江湖大数据产业园建设。推动先进装备制造、节能环保、生物制药和装配式建筑等产业发展。力争高新技术产业增加值增长15%。办好第五届矿博会、第五届湘南投洽会、第三届特色农博会，承办好全国篮球锦标赛等大型体育赛事，同步提升会展经济的质与量。加快发展软件开发、研发设计、检验检测、数据处理及网络设计研发等服务外包产业。实施“郴品网上行”工程，加快东谷等电商产业园建设，深化与阿里、京东等企业合作，引进培育一批电商企业和平台，大力发展分享经济，推进国家电子商务示范市建设。加强物流基础设施建设，提升智慧物流水平，深化物流服务管理创新，着力培育壮大物流市场主体，推进现代物流业加快发展。着力推进园区经济提质增效。完善园区基础设施和生产生活服务配套，提高园区综合承载力。完善园区发展质效考核评价体系，突出创新驱动、规模工业、财税贡献等指标考核，提高园区发展质量和效益。力争郴州经开区、资兴经开区创建国家级经开区取得突破性进展，加快永兴、宜章经开区申报国家级经开区前期工作。着力推进实体经济振兴发展。坚持“一企一策”、重点帮扶，帮助实体企业解决实际困难。在对企业“放水养鱼”的基础上，培植和拓展税源，增强财政“造血”功能。支持中、省驻郴企业“三供一业”分离移交。大力鼓励支持非公经济发展。建立“亲”“清”新型政商关系，在全社会形成尊重、关心、支持企业家的良好氛围。激励引导广大企业家坚定信心、奋发有为，提升素质、勇担责任，做爱国敬业、守法经营、创业创新、回报社会的表率。

（二）坚持多措并举，扩大有效需求。深入推进“重点项目建设四年行动计划”，着力扩大有效投资、引导消费升级、做强对外贸易，以有效需求支撑经济稳中有进、稳中向好。着力扩大有效投资。全市拟安排总投资1亿元以上重大项目1230个，年度计划投资1745亿元。重点抓好郴州北湖机场、莽山水库、桂新高速等292个基础设施项目；郴州国际矿产宝玉石文化创意产业园、金贵银业2000吨白银提纯扩能、金旺铋系深加工材料基地、郴州卷烟厂易地技改、南方石墨浮选提纯及加工、沃特玛新能源汽车、翔龙通航产业园、旗滨光伏光电玻璃、飞天山文化旅游产业园、湖南长鹿国际旅游休闲度假区等532个产业项目；农村综合服务平台、城乡污水垃圾处理、贫困村基础设施、公共卫生和义务教育扩容等341个民生和社会发展项目。抓好黄长、茶常、桂遂等高速公路及赣郴永兴铁路、郴资永轨道交通、通用机场等一批重大项目前期工作。引导项目科学选址，创新项目供地方式，依法依规优先保障重点项目用地。深化投资项目审批制度改革，强化重大项目调度和监管，确保项目建设进度和质量。着力促进消费升级。深入推进“互联网+商贸流通”行动，发挥新消费引领作用，加快培育形成新供给、新动力。加强专项市场、农产品流通市场建设，实施边贸市场促进工程、县乡流通再造工程、快递下乡工程。大力发展农村电商，完善农产品产销对接体系，真正把农产品销售作为重大民生抓实抓好。坚持整体性搬迁与功能性改造相结合，加大“棚户区改造三年行动计划”推进力度，完善基础设施和公共服务配套，促进住房消费。深化医养结合，推进居家和社区健康养老服务。扶持家政服务等新消费业态发展。积极拓展、提升文化体育消费。加强商品质量一体化监管，强化消费维权，营造放心消费环境。着力搞活对外贸易。扎实抓好国家级郴州综合保税区建设，搭建省级外贸综合服务平台和园区外贸综合服务中心。发挥重点企业龙头带动作用，做大做强有色金属加工贸易。继续实施外贸“破零倍增”计划，让郴州更多的优势企业和特色产品走向世界。

（三）坚持内外联动，深化改革开放。结合推进“招商引资四年行动计划”，以全面深化改革开放释放和挖掘潜力，推动经济向更广空间、更高水平发展。抓实各项改革任务。深化投融资体制改革，用足用好国家政策，积极争取中省预算内资金、专项建设基金、发行企业债券，推进PPP模式落实，更好地激活社会资本。支持企业进入资本市场直接融资。争取全省电力体制改革率先在郴州实施。深化财税体制改革，全面推进预算绩效管理。深化户籍及相关配套制度改革，着力提高户籍人口城镇化率。积极争取并推进国家服务业综合改革试点。深化行政审批、商事登记、市场监管机制和通关通检一体化改革，全面推行“双随机一公开”制度，探索“多证合一”“一址多照”“一照多址”，促进投资贸易便利化。深化农村集体产权制度改革，加快构建新型农业经营体系。深化供销合作社综合改革、国企改革和环境资源价格、公共资源交易等改革。强化创新驱动发展。充分发挥现有国家级技术平台作用，加强与高等院校、科研院所和大型企业的产学研合作，组织实施有色金属、石墨精深加工技术攻关和成果转化，提高精深加工和综合利用能力。支持郴州国家级中小企业创业基地等公共服务平台建设，扶持中小微企业创新发展。建立在外郴籍知名专家库，健全高层次创新人才培养和引进机制，积极开展柔性引才引智，探索发展“飞地实验室”“飞地研发中心”。深化与固高科技（香港）有限公司合作，加快组建郴州产业创新研究院，大力发展智能制造。积极创建国家知识产权示范城市。坚持质量兴郴，健全质量标准体系、质量安全保障体系和质量诚信体系。提高招商引资实效。坚持精准承接、精准对接，按照“五定”“五有”要求，统筹抓好产业链招商、节会招商、小分队招商。更好地发挥异地商会作用，深入推进“引老乡、回故乡、建家乡”活动。精心办好第二届郴商大会。强化签约项目跟踪落实，提高项目履约率、资金到位率和开工率。提升开放合作水平。进一步完善提升现有开放平台，支持发展郴州至广州港铁海联运国际物流，做大做强铁路口岸。加快郴州电子口岸综合信息平台和跨境快速通关系统建设；争取申报设立京港澳高速宜章口岸作业区，布局进口肉类制品指定查验场、粤港直通车站、跨境电商监管中心，争创湖南省跨境贸易电子商务服务试点城市。深化与“珠三角”“长三角”城市合作，探索发展“飞地经济”。

（四）坚持城乡统筹，推进新型城镇化。围绕“把郴州建设成为实力更强、活力更足、气质更美、开放度更高、品牌形象更好的现代化宜居城市，打造郴州城市升级版”的目标，以人的城镇化为核心，更加注重产城融合，更加注重城乡公共服务均等化，更加注重环境健康宜居和历史文化传承。健全城乡规划体系。坚持全域规划，推进经济社会发展规划、城市规划、土地利用规划等“多规合一”，构建更加科学的城乡空间结构体系。加快修改城市总体规划，完善地下综合管廊、轨道交通等专项规划，力争郴州“大十字”城镇群体系规划获得批复。进一步加强县城及乡村规划工作。严格规划执法，维护规划权威。完善城乡基础设施。市城区拟安排基础设施建设项目147个，年度计划投资80亿元。重点推进东江引水工程、市“四馆一厅”、高铁西站扩容提质、人民东路延伸段、地下综合管廊、“海绵城市”等项目。加大下湄桥片区提质改造力度。加快市城区立体人行过街设施、公共集约化停车设施建设。继续抓好“两供两治”，加快“气化郴州”步伐。建设干线公路25条500公里。持续抓好公路“治超”和“四好农村路”建设。加快耒水航道苏仙段改造。加强农村信息、电力、农田水利等基础设施建设和全市饮用水安全保障。强化城市精细管理。深化城管体制改革，强化环卫保洁、公厕管理、园林绿化、市政养护、渣土运输等精细管理，巩固“创卫”“创园”成果，深入抓好“创文”工作。持续抓好查违拆违控违，确保违法建设“零增长”。优化路网结构和交通组织，系统推进交通堵点整治，有效缓解拥堵问题。加快创建“公交都市”。打造特色精品城镇。推进“大十字”城镇群率先发展。着力提升县城承载能力。支持桂阳县撤县设市。抓好沙田、流峰、热水、龙海等全国建制镇示范试点、省级新型城镇化试点建设。突出抓好柏林、塘村、沤江等首批11个产镇融合示范镇、特色小镇建设。加快美丽乡村建设。扎实推进以农村环境整治、规范农村村民建房等为主要内容的“洁净乡村四年行动”。系统推进山水林田湖生态保护修复。加强古村落保护，传承历史文化。支持农村集体经济发展。努力让村民更富、村容更美、村风更好、村庄更宜居。

（五）坚持综合施策，提升生态品质。全面落实“一保二促三抓实”的环境保护工作总体要求，强化依法保护、综合施策、标本兼治，规划建立和严格保护生态绿线，推动建立横向生态补偿制度，严格重大环境问题责任追究，牢牢守住生态红线。抓实“蓝天”工程。落实大气污染防治行动计划，重点实施工业废气污染防治、城市扬尘污染控制、餐饮油烟整治全覆盖等十大工程，加快重点行业污染治理。积极创建空气质量达标示范城市。抓实“碧水”工程。全面推行“河长制”，坚持问题导向、因地制宜，实行一河一策、一湖一策，推进依法治水管水。抓好湘江保护和治理第二个“三年行动计划”，全面推进“一湖两河三江”生态综合治理。实施最严格的水资源管理制度，重点抓好水资源保护、雨洪水资源化利用、水环境整治等六大工程。认真抓好国务院批复的《东江湖风景名胜区总体规划》实施。抓实“净土”工程。积极推进生态红线制度建设，加强国土空间用途管控，严格落实主体功能区制度。持续推进重金属污染治理，组织开展土壤污染状况详查工作，加快受污染基本农田、工业场地土壤治理修复，抓好三十六湾等重点区域综合治理。抓实“绿地”工程。启动森林城市群建设，重点实施城区周边和主要交通干线面山绿化、森林景观提质改造、“裸露山地”绿化、天坑治理（二期）等国土绿化精品工程，让“林中之城”更加靓丽。

（六）坚持精准施策，加快脱贫攻坚。按照“四个切实”“五个一批”“七个精准”要求，全面落实精准扶贫、精准脱贫主体责任，充分发挥政府的主导作用、群众的主体作用，精准发力、攻坚克难，确保完成年度脱贫任务。积极稳妥推进易地搬迁。尊重群众意愿，落实政策要求，科学规划选址，坚持“六个结合”，扎实推进易地扶贫搬迁，加强易地搬迁对象后续帮扶，确保搬得出、稳得住、能致富、可持续。因地制宜发展特色产业。建立健全到户到人的精准扶持机制，坚持“四跟四走”，发挥农民合作组织、龙头企业作用，因地制宜推动每个贫困县、乡、村形成特色产业和拳头产品。突出191个全国乡村旅游扶贫重点村，抓实“乡村旅游扶贫八大行动”。切实抓好金融扶贫，力争小额扶贫信贷累计超过20亿元。积极争取生态补偿资金支持，扩大林业贴息贷款覆盖面。扶教助学强化教育扶贫。坚持扶贫先扶智，整合财政存量资金，补齐贫困县义务教育“短板”，特岗教师计划优先满足贫困县需要。完善建档立卡贫困家庭学生的教育资助体系，凡在公办普通高中就读的一律免除学杂费，属“两后生”的助其免费完成职业教育。加强就业技能培训，推进“一户一产业工人”全覆盖。精准“兜底”抓实社会保障。把经认定符合条件的“兜底”对象，全部纳入社保“兜底”。探索贫困人口医疗救助兜底保障办法，将建档立卡贫困人口纳入重特大疾病医疗救助范围，统筹合作医疗、医疗救助、临时救助和慈善救助。在全面建成小康社会的道路上，决不让一个贫困村落伍，决不让一名贫困群众掉队！

（七）坚持共建共享，促进社会和谐。始终把人民的利益放在首位，把群众的冷暖放在心上，保障人民共享改革发展成果。坚持创业带动就业。把稳定就业摆在更加突出的位置，落实更加积极的创新创业政策，完善创新创业公共服务平台，加强创新创业技能培训，统筹抓好城镇新增劳动力和农村转移劳动力就业。新增城镇就业6万人。提升社会保障水平。全面推进机关事业单位养老保险制度改革，继续抓好全民参保登记工作。推进农村低保标准和贫困标准两线融合。加强社会救助和农村留守老人、留守儿童关爱保护工作。完善社会求助体系。推进健康城市建设。把人民健康放在优先发展战略地位，大力普及健康生活、优化健康服务、完善健康保障、建设健康环境、发展健康产业。完善公共卫生和医疗服务体系，扎实推进“市城区医疗卫生服务发展三年行动计划”。深化城市公立医院综合改革。全面实施大病保险制度和药物保障供应制度。抓好重点传染病预防控制。加强出生缺陷防治。大力发展中医药事业。加快市公共卫生服务中心、市康养中心建设，启动市残疾人康复中心建设。统筹社会事业发展。扎实推进“中心城区化解‘大班额’三年行动计划”。统筹城乡教育资源配置，办好学前教育，推进义务教育均衡发展，进一步提升基础教育质量。加快职业教育转型发展。支持湘南学院和郴州职业技术学院、湘南幼专、郴州技师学院发展。完善终身教育体系，建设学习型社会。加快推进老年大学建设。坚持计划生育基本国策，改革完善计划生育服务管理。扎实推进文化强市建设，基本建成现代公共文化服务体系。坚持以人民为中心，推出更多文艺精品力作。加强文物和非物质文化遗产保护和利用。依法加强网络空间治理，净化网络环境。大力发展体育事业和体育产业，促进群众体育和竞技体育全面发展、全民健身和全民健康深度融合。夯实统计基层基础，提高依法统计水平。抓好村委会和社区居委会换届工作。认真做好广播影视、新闻出版、民族宗教、外事侨务、贸促、海关、检验检疫、文物、档案、气象、地震、水文、科普、地方志、慈善和妇女儿童、老年人、残疾人等工作。维护社会和谐稳定。大力培育和践行社会主义核心价值观，营造崇德向善、健康向上的社会氛围。创新社会治理体制机制，健全社会治安立体化防控体系，依法打击各类违法犯罪。严格落实安全生产责任制，推进安全生产形势持续稳定向好。强化食品药品监管，确保食品药品安全。深化信访制度改革，有效化解社会矛盾。强化公共应急管理，增强应急救援能力。加快创建国家社会信用体系建设示范城市，提高全民诚信水平。加强政府法律援助，维护社会公平正义。大力支持国防和军队改革，加强国防教育和动员、民兵预备役、人民防空、国家安全工作。扎实开展新时期双拥共建工作，继承和发扬“半床棉被”精神，严格落实优抚政策，推动军民融合深度发展，让军政军民团结的鱼水深情世代相传。

（八）坚持不忘初心，加强政府自身建设。牢记根本宗旨，强化“四个意识”，忠诚履职、务实担当，以为民的实举、发展的实绩、廉洁的实效，不断提高群众的满意度。坚持依法行政不动摇。始终忠诚于宪法和法律，将依法行政贯穿于政府工作的各个方面、各个环节。认真执行市人大及其常委会的决议决定，认真办理人大代表建议和政协提案，自觉接受市人大的法律监督、工作监督和市政协的民主监督，自觉接受其它各方面的监督。全面落实行政执法责任制，严格、规范、公正、文明执法。坚持科学理政不折腾。坚持和完善重大行政决策公众参与、专家论证、风险评估、合法性审查和集体讨论决定制度，主动听取民主党派、工商联、无党派人士和人民团体的意见建议，支持统战部门开展“凝心聚力‘十三五’行动”，不断提高科学民主决策水平。紧盯企业和群众需求，突出问题导向，创新政务服务方式，推进行政审批标准化建设，强化“三清单一目录”动态管理，全面清理规范行政审批中介服务，以壮士断腕的决心把“放管服”改革推向纵深。坚持从严治政不懈怠。严格落实全面从严治党“两个责任”，认真贯彻执行“两准则三条例”等各项党内法规，加强和规范党内政治生活。健全廉洁风险防控体系，强化权力运行监督，从严整治“懒散庸”，驰而不息纠“四风”，严肃查处违法违纪案件。全体政府工作人员特别是各级领导干部，要坚持围绕中心、狠抓落实，充满昂扬的斗志，充满爱民的情怀，努力建设办事的政府、高效的政府、廉洁的政府。

各位代表！唯有真抓实干，才能取信于民。2017年，我们要扎实抓好上述八个方面的工作，确保实现经济社会发展预期目标。在此基础上，还要撸起袖子加油干，着力办成一批有利于长远发展、有利于人民群众得实惠的大事实事。一是确保完成8件具有长远影响的大事。①成功创建全国文明城市；②东江引水（一期）工程竣工供水；③组建郴州产业创新研究院；④高铁西站扩容提质全面竣工；⑤京港澳高速郴州北互通竣工通车；⑥全面完成房地产去库存任务；⑦市“四馆一厅”竣工投入使用；⑧岳汝高速公路与广东段全面对接通车。二是全力做好5个具有长远影响的重大基础设施项目的前期工作，力争全面启动。①郴州北湖机场和通用机场建设；②东江引水（二期）工程建设；③南航郴州通航学院校区建设；④京广铁路郴州货运北站搬迁；⑤桂新高速公路建设。三是扎实抓好“十大惠民工程”。①扶贫惠民工程：完成易地扶贫搬迁3.26万人、10万贫困人口脱贫。②教育惠民工程：建设义务教育合格学校50所、农村公办幼儿园20所，补充乡村教师1800名、培训3800名，建设农村教师周转宿舍500套；市城区新增义务教育学位2万个、免收高中阶段学费。③健康惠民工程：实施残疾儿童抢救性康复300人、农村适龄妇女“两癌”免费检查4.97万人、孕产妇产前筛查4万人，建设强制戒毒所4所。④社保惠民工程：新增养老服务床位726张，职工参加医疗互助13.5万人。⑤安居惠民工程：完成大中型水库移民避险解困搬迁3000人、棚户区和农村危房改造3.7万户。⑥设施惠民工程：改造农村公路795公里、危桥40座，完成安保设施建设640公里；市城区建设（改造）、疏浚供水管网55公里；完成60个行政村配电网改造；新增管输天然气用户2.5万户。⑦水利惠民工程：完成62条河流、43座水库综合治理；农村饮水安全巩固提升受益30万人。⑧环境惠民工程：建设美丽乡村示范村11个、规范农村集中建房示范点22个、村庄整治示范村100个、乡村文化小广场364个；抓好295个公园游园管护；建设城乡垃圾收转运体系11个、污水管网47公里；柴油公交车尾气达标率80%以上。⑨信息惠民工程：建设364个4G网络站点和300个移动宽带站点。⑩服务惠民工程：建设（改造）农村综合服务平台350个以上，建设贫困地区文化综合服务中心示范点18个；完善市民乘坐公交车保险机制，让市民享有乘车保险；开展集贸市场及医疗机构计量器具免费检定。

各位代表！新蓝图任重道远，新使命催人奋进，新征程始于足下。让我们紧密团结在以习近平同志为核心的党中央周围，按照省第十一次党代会的总体部署和要求，在中共郴州市委的坚强领导下，全面落实“创新引领、开放崛起”和“产业主导、全面发展”战略，进一步强化“郴州担当”、凝聚“郴州力量”、凸显“郴州实效”，为加快建设“五个郴州”、打造湖南“新增长极”、率先全面建成小康社会而努力奋斗！
